# 

**Published:** 1886-06

**Authors:** 


Page.

ORIGINAL COMMUNICATIONS:

The Origin, Development, etc., of Tubercle of Tuberculosis. H. D. Schmidt ... 489
Paralysis Agitans. E. C. Mann ... 517
Lymphadenoma. J. W. Nelson ... 525

EDITORIALS:

Chicago Medical Society ... 534
The Medico-Legal Society of Chicago ... 534
Chicago as a Medical Center ... 535

SOCIETY REPORTS:

Transactions of the Chicago Gynæcological Society. *Meeting of March 19th.*
Presentation of Instruments and Pathological Specimens by W. W. Jaggard ... 536
Discussion on Pelvic Cellulitis ... 539
Epithelioma of the Uterus. De Laskie Miller ... 551
CHICAGO MEDICAL SOCIETY. *Meeting April 19th.*
Female Physicians as Residents in Insane Asylums. G. C. Paoli ... 553
Impermeable Stricture of the Urethra and Electrolysis. W. T. Belfield ... 554
Osteo-Plastic Resection. C. Fenger ... 560
The Annual Meeting of the Illinois State Medical Society ... 563
The Annual Meeting of the American Medical Association ... 564
The American Surgical Association ... 567
The American Laryngological Association ... 567
The Association of American Medical Editors ... 567
THE NINTH INTERNATIONAL MEDICAL CONGRESS. Meeting of the Executive Committee ... 568
List of General Officers and Presidents of Sections ... 570

BOOK REVIEWS:

Diagnosis of Diseases of the Brain and Spinal Cord. W. R. Gowers ... 573
A Guide to Examination of Urine. J. Tyson ... 574

BOOKS RECEIVED ... 575

INDEX ... 577

